# Effects of the probiotic *Lactiplantibacillus plantarum* IMC 510® on body composition, biochemical parameters, gut microbiota composition and function, and clinical symptoms of overweight/obese subjects

**DOI:** 10.3389/fnut.2023.1142527

**Published:** 2023-04-11

**Authors:** Giuditta Pagliai, Maria Magdalena Coman, Simone Baldi, Monica Dinu, Giulia Nannini, Edda Russo, Lavinia Curini, Barbara Colombini, Sofia Lotti, Marco Pallecchi, Leandro Di Gloria, Gianluca Bartolucci, Matteo Ramazzotti, Maria Cristina Verdenelli, Francesco Sofi, Amedeo Amedei

**Affiliations:** ^1^Department of Experimental and Clinical Medicine, University of Florence, Florence, Italy; ^2^Synbiotec S.r.l, Camerino, Italy; ^3^Department of Neurosciences, Psychology, Drug Research and Child Health, University of Florence, Florence, Italy; ^4^Department of Biomedical, Experimental and Clinical Sciences “Mario Serio”, University of Florence, Florence, Italy; ^5^Unit of Clinical Nutrition, Careggi University Hospital, Florence, Italy; ^6^Interdisciplinary Internal Medicine Unit, Careggi University Hospital, Florence, Italy

**Keywords:** *Lactiplantibacillus plantarum IMC 510®*, probiotic supplementation, gut microbiota, obesity, clinical trial

## Abstract

**Background and aim:**

In recent decades, obesity prevalence has reached epidemic proportions and considering the pivotal role of gut microbiota (GM) in the regulation of energy balance, alternative non-pharmacological approaches involving probiotics’ administration have been proposed. The aim of the present study was to evaluate the effect of *Lactiplantibacillus plantarum* IMC 510® supplementation on anthropometric and biochemical parameters, GM composition and functionality, and gastrointestinal and general symptoms of overweight/obese subjects.

**Methods:**

Forty overweight/obese subjects were randomly assigned to daily consume the probiotic *Lactiplantibacillus plantarum* IMC 510® or placebo for 3 months. Before and after the administration period, anthropometric and biochemical parameters, self-administered questionnaires, and plasma and stool samples were obtained from each participant. The GM characterization was performed with 16S rRNA sequencing, while fecal short (SCFAs) and medium (MCFAs) chain fatty acids were analyzed with a gas chromatography–mass spectrometry protocol.

**Results:**

Compared to placebo, probiotic supplementation determined a significant decrease in body weight, BMI, waist circumference, waist-to-height ratio, and blood glucose. Moreover, probiotic administration produced a significant decrease of the genera *Hafnia-Obesumbacterium* and *Romboutsia* and an increase of *Succiniclasticum* spp.; conversely, placebo administration resulted in the decrease of Actinomycetaceae and an increase of both *Alloprevotella* spp. and of the levels of pro-inflammatory hexanoic and heptanoic acids.

**Conclusion:**

Thanks to its effect in increasing some beneficial gut bacteria and lowering effects on waist circumference, fasting glucose levels and gastrointestinal symptoms of obese subjects, *Lactiplantibacillus plantarum* IMC 510® supplementation could represent a future and encouraging strategy for the prevention or treatment of obesity.

## Introduction

In recent decades, obesity prevalence has reached epidemic proportions and novel advances allowed a better characterization of its etiology, highlighting the potential contribution of factors not usually considered to be involved in changes in energy balance and body composition (1, 2). Indeed, experimental and clinical studies have demonstrated the role of the gut microbiota (GM) in the regulation of energy balance and the occurrence of excess body weight (3).

In this regard, it is widely accepted that high microbial diversity is fundamental for the maintaining of both host immune homeostasis and a balanced fermentable ability of the non-digestible dietary components, which is crucial for short-chain fatty acids (SCFAs) production (4); nevertheless, it has been reported that lean and obese subjects exhibit different GM compositions (5). In detail, recent findings have documented an imbalanced *Firmicutes/Bacteroidota* ratio in obese subjects, in addition to an increase in the Actinobacteria phylum and a decrease in *Verrucomicrobiota* (6). Moreover, compared to lean subjects, obese individuals revealed significantly reduced levels of *Bacteroidota* members and *Clostridium perfringen*s (7), while Christensenellaceae members have been proposed as a microbial biomarker for obesity (8) and both *Bacteroides thetaiotaomicron* and *Methanobrevibacter smithii* were reported to enhance adipose tissue accumulation (9).

On the other hand, a study conducted by Million and colleagues on 68 obese subjects and 47 controls aiming to characterize the obesity-associated gut microbiota documented that lean subjects revealed higher levels of *Lactobacillus paracasei* and *Lactobacillus plantarum*, whereas *Lactobacillus reuteri* was highly represented in obese individuals (10).

Overall, during the past 25 years, more than 120 drugs have been studied for the treatment of obesity, but only very few have been approved and maintained on the market (11). In addition, although exercise and diet are the first-line interventions to correct overweight, obesity, and related metabolic diseases, they often experience failure or poor results (12). However, considering the evidence highlighting the pivotal GM role in nutrient acquirement, energy harvest, and host metabolic pathways modulation, there is currently a growing interest in alternative and effective short-term non-pharmacological approaches to weight control, that involve the use of natural ingredients (13). For instance, the oral administration of probiotics has been proposed as a viable way to modulate the GM thus promoting weight reduction. However, although is well established that microbial metabolites, especially SCFAs which can enter the bloodstream and reduce appetite and energy intake, and specific GM bacteria can reduce diet-induced obesity through increased energy expenditure, the mechanisms by which probiotic supplementation can affect the GM of overweight or obese individuals are still largely unknown (14). Nevertheless, our preliminary data showed that the supplementation of a novel probiotic formulation based on *Lactiplantibacillus plantarum* IMC 510® determined a significant reduction in weight and food intake in obese rats and body weight, body mass index (BMI) and waist circumferences in a restricted cohort of overweight/obese subjects (15, 16).

Therefore, the present randomized controlled trial aimed to evaluate the effect of a new probiotic formulation based on *Lactiplantibacillus plantarum* IMC 510® on anthropometric and biochemical parameters, GM composition and function, and gastrointestinal and systemic clinical manifestations of overweight/obese subjects.

## Materials and methods

### Study population

A total of 40 subjects (20 males and 20 females) with a mean age of 51.6 ± 13.2 years were enrolled in the study. Participants were considered eligible for inclusion if they were aged between 18 and 65 years and had a BMI ≥ 25 kg/m^2^. Exclusion criteria were as follows: use of antibiotics or continued use of probiotics in the 2 months before enrolment; use of other treatments (medications or nutritional programs) that affect body weight, food intake, and/or energy expenditure; pregnancy or breast-feeding; and enrolment in another obesity treatment program.

The flow of participants through the trial according to the CONSORT (CONsolidated Standards of Reporting Trials) diagram is presented in Supplementary Figure 1.

### Study design

The present study is a randomized, double-blinded parallel controlled trial. Before the randomization, there was a 2-week run-in period to assess participants’ commitment, motivation, and availability. After the run-in period, eligible participants were randomly divided into two groups, each assigned to consume the probiotic *Lactiplantibacillus plantarum* IMC 510® or placebo for 3 months. During the intervention period, all participants were encouraged not to alter their lifestyle, exercise, or eating habits. Study procedures were approved by the Ethics Committee of the Tuscany Region, Careggi University Hospital (14592_spe), registered at clinicaltrials.gov (identifier: NCT05358301), and adhered to the principles of the Declaration of Helsinki and the Data Protection Act.

### Data collection

Data collection and follow-up measurements were performed at the Clinical Nutrition Unit of Careggi University Hospital. After an overnight fast, all participants were examined between 6:30 AM and 9:30 AM. General information about demographics, education, personal medical history, and use of drugs was collected from each participant at the beginning of the study. Habitual dietary intake was assessed using a 3-day food diary recorded over 2 weekdays and 1 weekend day at the baseline and after 3 months of intervention, while compliance was evaluated during the follow-up visit by counting empty boxes of capsules on return. Moreover, validated self-administered questionnaires were collected from each participant at the beginning and the end of the intervention period.

The questionnaires included a modified form of the Global Assessment of Improvement Scale (GAI) and the Symptom Severity Scale (SSS). The GAI assesses the improvement of symptoms using a 7-point scale, with higher scores meaning an improvement in the symptoms. The severity of abdominal pain, bloating, stool consistency, headache, fatigue, nausea, concentration, muscle pain, and quality of life have been investigated in response to the following question: “Compared to the way you felt before you entered the study, have your symptoms over the past seven days been:” (1) “Substantially Worse,” (2) “Moderately Worse,” (3) “Slightly Worse,” (4) “No Change,” (5) “Slightly Improved,” (6) “Moderately Improved,” or (7) “Substantially Improved.” The SSS is a multidimensional rating scale assessing overall symptom severity on a visual analog scale (VAS). An overall score has been calculated from six items: abdominal pain severity, abdominal pain frequency, bloating, bowel satisfaction, abdominal heaviness, and quality of life. The modified SSS ranges from 0 to 600, with higher scores meaning more severe symptoms.

Body weight and body composition were measured at each clinical evaluation. Weight and height were measured using a stadiometer. BMI was calculated as the weight (kg)/height (m^2^), while WHtR (waist-to-height ratio) was calculated as waist circumference (cm)/height (cm). Participants were classified as overweight if their BMI was ≥25 kg/m^2^, and < 30 kg/m^2^, while obese if their BMI was ≥30 kg/m^2^. Body composition was determined by a bioelectrical impedance analysis device (AKERN, model 101 Sport Edition).

At baseline and the end of each intervention phase, in addition to fecal samples for the analysis of GM and SCFAs, also venous blood samples were collected in evacuated plastic tubes (Vacutainer, Becton Dickinson). Blood samples were centrifuged at 3,000 rpm for 15 min (4°C) and stored in aliquots at −80°C until further analyses. Total cholesterol and its subtypes, triglycerides, glucose, insulin, serum electrolytes, standard liver panel enzymes, and mineral and vitamin profiles were measured according to conventional laboratory standard methods.

### Interventions

The experimental probiotic food supplement used in the present clinical study is produced by Synbiotec S.r.l. (Camerino, Macerata, Italy). Each capsule contains the probiotic strain *Lactiplantibacillus plantarum* IMC 510® at a concentration of 15 billion live cells (CFU/capsule). The assumption is of N = 1 capsule/day, preferably at breakfast, for 3 months. The capsules can be opened, and their content can be dispersed in a cold liquid or semi-solid food. The capsules should be stored at room temperature in the original container or a clean covered container, out of the reach of children. When stored in a dry, clean environment, out of direct sunlight, the probiotic product has a shelf-life of at least 24 months. Samples of probiotic supplements in closed and sealed boxes have been provided to participants at the beginning of the study, in sufficient quantities for the entire duration of the study (90 capsules for 3 months).

The placebo food supplement (i.e., capsules without added probiotics) was administrated as N = 1 capsule/day with the same intake modalities as the experimental probiotic. Probiotic and placebo capsules appeared to be identical to ensure the blindness of both physicians and participants.

### Characterization of gut microbiota

Total DNA was extracted using the DNeasy PowerSoil Pro Kit (Qiagen, Hilden, Germany) from frozen (−80°C) stool samples, according to the manufacturer’s instructions. Briefly, 0.25 g of stool samples were added to a bead-beating tube and homogenized with TissueLyser LT (Qiagen, Hilden, Germany) for 5 min at 50 Hz. Afterwards, DNA was captured on a silica membrane in a spin column format, washed, and eluted. The quality and quantity of extracted DNA were assessed with both NanoDrop ND-1000 (Thermo Fisher Scientific, Waltham, United States) and Qubit Fluorometer (Thermo Fisher Scientific, Waltham, United States) and then it was frozen at −20°C. Subsequently, genomic DNA samples were sent to IGA Technology Services (Udine, Italy) where amplicons of the variable V3–V4 region of the bacterial 16S rRNA gene, delimited through the primers 341F and 805R, were sequenced in paired-end (2 × 300 cycles) on the Illumina MiSeq platform, according to the Illumina 16S Metagenomic Sequencing Library Preparation protocol.

Demultiplexed sequence reads were processed using QIIME2 2022.8 (17). The sequencing primers and the reads without primers were removed using the Cutadapt tool. DADA2 (18) was used to perform paired-end reads merging, filtering, and chimeras removal steps after trimming nucleotides from both forward and reverse reads based on the quality profiles (−-p-trunc-len-f 264 and --p-trunc-len-r 179) (Supplementary Table 1). Hence, ASVs (amplicon sequence variants) were generated and the taxonomic assignments have been performed through Scikit-learn Bayesian Classificator retrained on the V3-V4 16S region of SILVA 138.

### Analysis of fecal SCFAs and MCFAs by gas chromatography–mass spectrometry

The qualitative and quantitative evaluation of fecal SCFAs and MCFAs (medium-chain fatty acids) was performed by Agilent gas chromatography–mass spectrometry (GC–MS) system composed of 5,971 single-quadrupole mass spectrometer, 5,890 gas chromatograph, and 7,673 autosampler, through our previously described GC–MS method (2).

Briefly, just before the analysis, stool samples were thawed and added with sodium bicarbonate 0.25 mM solution (1:1 w/v) in a 1.5 ml centrifuge tube. Then, the obtained suspensions were sonicated for 5 min, centrifuged at 5,000 rpm for 10 min and then the supernatants were collected. The SCFAs and MCFAs were finally extracted as follows: an aliquot of 100 μl of sample solution (corresponding to 0.1 mg of stool sample) was added of 50 μl of internal standards mixture, 1 ml of tert-butyl methyl ether, and 50 μl of HCl 6 M + 0.5 M NaCl solution in a 1.5 ml centrifuge tube. Subsequently, each tube was shaken in a vortex apparatus for 2 min, centrifuged at 10,000 rpm for 5 min, and lastly, the solvent layer was transferred to an autosampler vial and processed three times.

### Statistical analysis

Statistical analysis of anthropometric and biochemical parameters was performed using the SPSS (Chicago, IL, United States) software for Macintosh (Version 23.0). Data were reported as mean ± standard deviation (SD) or number and percentage, as appropriate. The non-parametric Mann–Whitney test was used for comparisons between intervention and control groups. The χ2-test was used to test for proportions. To compare the effect of the two different treatments, a general linear model was performed for repeated measurements. Data for the general linear model were reported as geometric mean and 95% confidence interval. A value of *p* <0.05 was considered to indicate statistical significance.

Regarding bacterial communities, statistical analyses were performed in R 4.2.1 with the help of the packages phyloseq 1.4.0, DESeq2 1.36.0 and other packages satisfying their dependencies as vegan 2.6–2. Packages ggplot2 3.3.5, ggh4x 0.2.2, and dendextend 1.15.2 were used to plot data and results. A rarefaction analysis on ASV was performed on every sample using the function rarecurve (step 100 reads), further processed to highlight saturated samples (arbitrarily defined as saturated samples with a final slope in the rarefaction curve with an increment in ASV number per reads <1e-5) to check the sample ASV saturation. Data were reported as mean ± standard deviation (SD), number and percentage, or median percentage and interquartile range (IQR), as appropriate. Shannon index, Observed ASV richness, and Pielou’s evenness were used to estimate bacterial diversity in each sample using the function estimate_richness from phyloseq. The Pielou’s evenness index was calculated using the formula E = S/log(R), where S is the Shannon diversity index and R is the number of ASVs in the sample. Differences in all indices were tested using the Wilcoxon test. PCoAs were performed on proportional count data of each sample, adjusted with square root transformation. At the different taxonomic ranks, the differential analyses of the abundances have been computed through DESeq2 on raw count data.

Furthermore, the software GraphPad Prism (v.5) was used for the statistical analysis of the fecal SCFAs’ and MCFA’s levels; pairwise differences between groups were assessed using Wilcoxon signed-rank test and value of *p* values less than 0.05 were considered statically significant.

## Results

### Baseline characteristics of the study population

Baseline characteristics of the study population, according to the randomization, are shown in Table 1. No significant differences were reported between subjects randomized to consume probiotic or placebo capsules, apart from smoking habits and body weight at the baseline. No smokers were present in the probiotic group, while 35.3% of the placebo group were smokers (*p* = 0.025); furthermore, subjects in the probiotic group started with higher body weight than subjects in the placebo group (90 ± 10.4 kg vs. 82.4 ± 16.2 kg, *p* = 0.038, respectively). Baseline levels of LDL-cholesterol appeared to be above optimal levels in both groups. All other biochemical parameters were within normal ranges.

**Table 1:** Baseline characteristics of the study population according to the randomization.

Variable	All (*n* = 40)	Probiotic (*n* = 20)	Placebo (*n* = 20)	*p*-value
Age, y	51 ± 13.5	51 ± 12.7	51 ± 14.7	0.946
Female sex, n (%)	20 (50)	9 (52.9)	11 (47.1)	0.999
Weight, Kg	86.1 ± 14	90 ± 10.4	82.4 ± 16.2	**0.038**
BMI, Kg/m^2^	29.7 ± 4.2	30.8 ± 3.8	28.7 ± 4.5	0.085
Waist circumference, cm	101.6 ± 12.9	104.7 ± 13.1	98.5 ± 13.9	0.131
WHtR	0.6 ± 0.1	0.6 ± 0.1	0.6 ± 0.1	0.092
Smoking habit, n (%)	6 (17.6)	0 (0)	6 (35.3)	**0.018**
Physical activity, n (%)	13 (38.2)	7 (41.2)	6 (35.3)	0.999
Fat mass, Kg	29.3 ± 9.6	31.4 ± 9.5	27.0 ± 9.5	0.208
Fat mass, %	33.8 ± 8.3	34.8 ± 9.3	32.6 ± 7.3	0.525
Fat-free mass, Kg	56.9 ± 11.4	58.6 ± 10.9	55.0 ± 11.9	0.257
Total body water, L	41.7 ± 8.3	42.9 ± 8	40.3 ± 8.7	0.257
White blood cells, ×10^9^/L	7.0 ± 2.0	6.5 ± 1.3	7.6 ± 2.5	0.099
Red blood cells, ×10^12^/L	4.9 ± 0.4	5.0 ± 0.4	4.8 ± 0.4	0.433
Hemoglobin, g/dL	14.7 ± 1.2	14.9 ± 1.3	14.4 ± 1.1	0.231
Glucose, g/L	97.3 ± 14.4	98.7 ± 16.7	95.9 ± 12.0	0.973
Urea, g/L	0.3 ± 0.1	0.3 ± 0.1	0.4 ± 0.1	0.191
Creatinine, mg/dL	0.9 ± 0.2	0.9 ± 0.2	0.8 ± 0.2	0.657
Sodium, mEq/L	141.6 ± 1.7	142 ± 1.6	141.2 ± 1.7	0.182
Potassium, mEq/L	4.6 ± 0.4	4.6 ± 0.3	4.6 ± 0.4	0.838
Calcium, mg/dL	9.4 ± 0.6	9.4 ± 0.8	9.4 ± 0.4	0.290
Magnesium, mg/dL	2 ± 0.1	2 ± 0.1	2 ± 0.1	0.946
AST, U/L	22.0 ± 10.9	19.0 ± 5	24.9 ± 14.3	0.160
ALT, U/L	23.0 ± 12.6	21.5 ± 9.2	24.6 ± 15.5	0.919
Triglycerides, mg/dL	125.2 ± 105.5	106.0 ± 45.8	144.3 ± 141.7	0.786
Total Cholesterol, mg/dL	194.4 ± 33.7	193.3 ± 29.4	195.6 ± 38.3	0.919
HDL-cholesterol, mg/dL	55.0 ± 13.6	56.6 ± 14.9	53.9 ± 12.5	0.786
LDL-cholesterol, mg/dL	116.1 ± 29.9	117.3 ± 24.9	114.7 ± 35.6	0.728
Uric Acid, mg/dL	5.2 ± 1.2	5.2 ± 1.1	5.3 ± 1.3	0.892
eGFR	84.3 ± 8.7	83.7 ± 7.6	84.9 ± 9.9	0.309
Total energy, kcal/die	1943 ± 560	2038 ± 545	1842 ± 576	0.281
Carbohydrate, % of energy	42.8 ± 7.2	43.6 ± 7.1	42.0 ± 7.3	0.626
Protein, % of energy	16.5 ± 3.5	16.8 ± 4.0	16.1 ± 3.1	0.999
Total fat, % of energy	40.2 ± 5.1	39.1 ± 5.1	41.3 ± 5.0	0.299
Saturated fat, % of energy	17.8 ± 13.1	19.1 ± 14.1	16.4 ± 12.3	0.626
Dietary cholesterol, mg/die	239.0 ± 127.8	253.7 ± 146.6	223.3 ± 107.1	0.830
Omega-3, g/die	1.2 ± 0.7	1.2 ± 0.6	1.3 ± 0.9	0.892
Dietary fiber, g/die	18.4 ± 7.3	21.3 ± 8.4	15.4 ± 4.3	0.054

Data are reported as mean ± standard deviation (SD), *p* value calculated using the Mann–Whitney test.

BMI, body mass index; WHtR, waist-to-height ratio; AST, aspartate aminotransferase; ALT, alanine aminotransferase; eGFR, estimated glomerular filtration rate. Statistically significant *p*-values are shown in bold.

### Intervention effects on body composition

Table 2 displays the changes in the anthropometric parameters after the intervention and control periods. No significant difference between the two groups was found. The group under probiotic supplementation revealed a significant decrease (*p* < 0.05) in body weight (−0.7 kg, *p* = 0.026), BMI (−0.2, *p* = 0.032), waist circumference (−0.9 cm, *p* = 0.043), and WHt ratio (−0.01, *p* = 0.041). Moreover, even if not statistically significant, but at the limit of significance, the probiotic group also showed a decrease in fat mass (−0.9 kg, *p* = 0.075).

**Table 2:** Effects of the probiotic and placebo on body composition.

Variable	Probiotic pre	Probiotic post	*p* value^†^	Placebo pre	Placebo post	*p* value^†^	Change Probiotic	Change Placebo	*p* value^°^
Weight, Kg	90 (84.6; 95.3)	89.3 (84.2; 94.5)	**0.026**	82.4 (74; 90.7)	82.5 (73.6; 91.3)	0.791	−0.7 (−1.2; −0.1)	0.1 (−0.7; 0.9)	0.215
BMI, Kg/m^2^	30.8 (28.8; 32.7)	30.5 (28.6; 32.4)	**0.032**	28.7 (26.4; 31)	28.7 (26.3; 31.2)	0.932	−0.2 (−0.4; 0)	0 (−0.3; 0.3)	0.269
Waist circumference, cm	104.6 (98.7; 110.5)	103.8 (97.9; 109.6)	**0.043**	98.5 (91.4; 105.7)	97.9 (90.4; 105.4)	0.135	−0.9 (−1.7; 0)	−0.6 (−1.4; 0.2)	0.916
WHtR	0.6 (0.6; 0.6)	0.6 (0.6; 0.6)	**0.041**	0.6 (0.5; 0.6)	0.6 (0.5; 0.6)	0.118	0 (0; 0)	0 (0; 0)	0.876
Fat mass, Kg	31.4 (26.3; 36.4)	30.5 (25.9; 35.1)	0.075	27 (21.5; 32.5)	27.3 (21; 33.5)	0.550	−0.9 (−1.9; 0.1)	0.3 (−0.8; 1.5)	0.164
Fat mass, %	34.8 (29.9; 39.8)	34.2 (29.5; 38.9)	0.157	32.6 (28.4; 36.8)	32.8 (28.2; 37.3)	0.757	−0.7 (−1.6; 0.3)	0.1 (−0.8; 1.1)	0.177
Fat-free mass, Kg	58.6 (52.8; 64.5)	58.9 (53.2; 64.5)	0.594	55 (48.1; 61.9)	54.8 (47.9; 61.8)	0.575	0.2 (−0.6; 1)	−0.2 (−0.8; 0.5)	0.479
Total body water, L	42.9 (38.6; 47.2)	42.6 (38.5; 46.7)	0.535	40.3 (35.2; 45.3)	40.1 (35.1; 45.2)	0.634	−0.3 (−1.5; 0.8)	−0.1 (−0.6; 0.4)	0.851

General linear model for repeated measurements. Data are reported as geometric mean and 95% confidence interval.

† *p* value calculated using the Wilcoxon signed-ranks test.

° *p* value calculated using the Mann–Whitney test. BMI = body mass index, WHtR = waist-to-height ratio. Statistically significant *p*-values are shown in bold.

### Impact of the intervention on biochemical parameters

Regarding the biochemical parameters, a general linear model for repeated measurements showed that the probiotic led to a significant decrease in blood glucose (−3.9 g/l; *p* = 0.033) and a significant increase in uric acid (0.2 mg/dl; *p* = 0.040) after 3 months of intervention. On the other hand, the placebo led to a significant reduction of white blood cells (−0.8; *p* = 0.006) and red blood cells (−0.1, *p* = 0.005), with a statistically significant difference between the two groups (*p* = 0.010) just for the white blood cells. No significant changes were observed for the other blood parameters (Table 3).

**Table 3:** Effect of the probiotic and placebo on biochemical parameters.

Variable	Probiotic pre	Probiotic post	*p* value^†^	Placebo pre	Placebo post	*p* value^†^	Change Probiotic	Change Placebo	*p* value^°^
White blood cells, × 10^9^/L	6.5 (5.8; 7.1)	6.5 (5.7; 7.3)	0.741	7.6 (6.3; 8.8)	6.8 (5.7; 7.9)	**0.006**	0.1 (−0.3; 0.5)	−0.8 (−1.2; −0.3)	**0.010**
Red blood cells, × 10^12^/L	5 (4.8; 5.2)	5 (4.7; 5.2)	0.625	4.8 (4.7; 5)	4.7 (4.5; 5)	**0.005**	0 (−0.1; 0.1)	−0.1 (−0.2; 0)	0.070
Hemoglobin, g/dL	14.9 (14.3; 15.6)	14.9 (14.2; 15.6)	0.605	14.4 (13.8; 15)	14.2 (13.5; 14.8)	0.073	−0.1 (−0.3; 0.2)	−0.2 (−0.5; 0)	0.161
Glucose, g/L	98.6 (90.1; 107.2)	94.8 (86.4; 103.1)	**0.033**	95.9 (89.7; 102.1)	97.5 (89; 105.9)	0.537	−3.9 (−7.4; −0.4)	1.6 (−3.7; 6.9)	0.084
Urea, g/L	0.3 (0.3; 0.4)	0.3 (0.3; 0.4)	0.973	0.4 (0.3; 0.4)	0.3 (0.3; 0.4)	0.484	0 (0; 0)	0 (−0.1; 0)	0.366
Creatinine, mg/dL	0.9 (0.8; 1)	0.9 (0.8; 1)	0.675	0.8 (0.8; 0.9)	0.8 (0.8; 0.9)	0.349	0 (0; 0.1)	0 (0; 0)	0.914
Sodium, mEq/L	142 (141.2; 142.8)	141.4 (140.9; 141.8)	0.173	141.1 (140.3; 142)	141 (139.8; 142.2)	0.802	−0.6 (−1.6; 0.3)	−0.1 (−1.1; 0.9)	0.540
Potassium, mEq/L	4.6 (4.4; 4.7)	4.4 (4.2; 4.5)	0.063	4.6 (4.4; 4.8)	4.6 (4.4; 4.7)	0.596	−0.2 (−0.4; 0)	−0.1 (−0.3; 0.2)	0.324
Calcium, mg/dL	9.4 (9; 9.9)	9.4 (9; 9.9)	0.928	9.4 (9.2; 9.6)	9.3 (9.1; 9.6)	0.399	0 (−0.1; 0.1)	0 (−0.2; 0.1)	0.728
Magnesium, mg/dL	2 (2; 2.1)	2 (2; 2.1)	0.083	2 (1.9; 2.1)	2 (2; 2.1)	0.332	0 (0; 0.1)	0 (0; 0.1)	0.708
AST, U/L	19.1 (16.5; 21.6)	20.4 (17.4; 23.4)	0.130	24.9 (17.6; 32.3)	23.2 (16.3; 30.1)	0.539	1.4 (−0.4; 3.2)	−1.8 (−7.7; 4.2)	0.466
ALT, U/L	21.5 (16.7; 26.2)	21.6 (16; 27.2)	0.935	24.6 (16.6; 32.5)	25.8 (16.2; 35.5)	0.456	0.1 (−2.9; 3.1)	1.2 (−2.2; 4.7)	0.904
Triglycerides, mg/dL	106 (82.5; 129.5)	99.9 (78.1; 121.7)	0.308	144.3 (71.5; 217.1)	123.3 (88.8; 157.8)	0.320	−6.1 (−18.3; 6.1)	−21 (−64.4; 22.4)	0.491
Total cholesterol, mg/dL	193.3 (178.2; 208.4)	194.2 (176; 212.5)	0.871	195.6 (175.9; 215.3)	193.3 (173.3; 213.3)	0.678	0.9 (−11.1; 13)	−2.3 (−13.8; 9.2)	0.718
HDL-cholesterol, mg/dL	56.1 (48.4; 63.7)	54.4 (47.2; 61.5)	0.122	53.9 (47.5; 60.4)	51.9 (45.8; 58)	0.116	−1.7 (−3.9; 0.5)	−2.1 (−4.7; 0.6)	0.945
LDL-cholesterol, mg/dL	117.3 (104; 130.6)	120.3 (101.7; 138.8)	0.604	114.7 (94.1; 135.3)	119.2 (97.3; 141)	0.310	3 (−8.9; 14.9)	4.5 (−4.7; 13.6)	0.950
Uric acid, mg/dL	5.2 (4.6; 5.7)	5.4 (4.8; 5.9)	**0.040**	5.3 (4.6; 6)	5.5 (4.8; 6.1)	0.281	0.2 (0; 0.4)	0.2 (−0.2; 0.6)	0.535
eGFR	83.7 (79.6)	82.6 (78.1; 87.2)	0.622	84.9 (79.8; 90)	84.6 (79.2; 90)	0.670	−1.1 (−5.6; 3.4)	−0.4 (−2.1; 1.4)	0.764

General linear model for repeated measurements. Data are reported as geometric mean and 95% confidence interval. ^†^p value calculated using the Wilcoxon signed-ranks test,

° *p* value calculated using the Mann–Whitney test.

AST, aspartate aminotransferase; ALT, alanine aminotransferase; eGFR, estimated glomerular filtration rate. Statistically significant *p*-values are shown in bold.

### Evaluation of the clinical symptoms

To assess the changes in gastrointestinal and systemic symptoms during the study period, participants were asked to report the improvement and severity of each different symptom using validated questionnaires. A general linear model for repeated measurement showed no significant changes in the total GAI and SSS scores in both probiotic and placebo groups. From the analysis of the individual items of the questionnaires, probiotic supplementation led to an unexpected variation in muscle pain (−8%; *p* = 0.030). In addition, probiotic supplementation led to a close to significant improvement in abdominal heaviness (−39.3%; *p* = 0.053) and the interference of gastrointestinal symptoms with quality of life (−44.7%; *p* = 0.064). On the other hand, a slight, not significant decrease in abdominal pain intensity (−61.4%; p = 0.064) and frequency (−58.9%; *p* = 0.067) was observed in the placebo group (Table 4).

**Table 4:** Effect of the probiotic and placebo supplementation on clinical symptoms.

Variable	Probiotic pre	Probiotic post	*p* value^†^	Placebo pre	Placebo post	*p* value^†^	Change Probiotic	Change Placebo	*p* value^°^
GAI	47.6 (42.5; 52.7)	47 (41.7; 52.3)	0.537	47.7 (42.7; 52.7)	47.9 (43.8; 52)	0.905	−0.6 (−2.6; 1.4)	0.2 (−2.9; 3.3)	0.551
Abdominal pain	5.8 (4.9; 6.6)	5.7 (4.8; 6.6)	0.875	5.7 (5.1; 6.3)	5.9 (5.3; 6.5)	0.508	−0.1 (−0.8; 0.7)	0.2 (−0.4; 0.7)	0.164
Bloating	4.8 (4.1; 5.6)	4.8 (3.9; 5.6)	0.750	5.2 (4.5; 5.8)	5.4 (4.8; 6.1)	0.361	−0.1 (−0.4; 0.3)	0.2 (−0.3; 0.8)	0.164
Stool consistency	5.6 (5; 6.1)	5.6 (4.9; 6.3)	0.999	5.4 (4.6; 6.2)	5.4 (4.4; 6.3)	0.750	0 (−0.7; 0.7)	−0.1 (−0.4; 0.3)	0.428
Fatigue	4.8 (4.2; 5.5)	5 (4.3; 5.7)	0.269	4.9 (4; 5.9)	5.1 (4.3; 5.9)	0.727	0.2 (−0.2; 0.5)	0.1 (−0.6; 0.8)	0.501
Nausea	5.9 (5; 6.7)	5.7 (4.8; 6.6)	0.188	5.7 (4.8; 6.6)	5.3 (4.4; 6.2)	0.203	−0.2 (−0.4; 0.1)	−0.4 (−1.1; 0.2)	0.864
Headache	5.4 (4.4; 6.3)	5.5 (4.5; 6.5)	0.668	5.2 (4; 6.3)	5.5 (4.6; 6.4)	0.231	0.1 (−0.5; 0.7)	0.4 (−0.2; 1)	0.473
Concentration	4.9 (4.1; 5.8)	4.9 (4.1; 5.7)	0.718	5.4 (4.6; 6.1)	5.2 (4.4; 6.1)	0.608	−0.1 (−0.4; 0.3)	−0.1 (−0.6; 0.4)	0.999
Muscle pain	5 (4.2; 5.8)	4.6 (3.4; 5.5)	**0.030**	4.8 (4; 5.7)	4.6 (3.8; 5.4)	0.332	−0.4 (−0.8; 0)	−0.2 (−0.7; 0.3)	0.352
Quality of life	5.4 (4.9; 5.9)	5.3 (4.7; 5.9)	0.332	5.4 (4.8; 6)	5.5 (4.9; 6.2)	0.579	−0.1 (−0.4; 0.1)	0.1 (−0.3; 0.6)	0.230
SSS	126.2 (69.6; 182.8)	85.3 (41.9; 128.7)	0.099	138.2 (89.9; 186.6)	124.1 (86.5; 161.7)	0.437	−40.9 (−90.4; 8.6)	−14.1 (−51.7; 23.4)	0.448
Abdominal pain	17.1 (3.1; 31)	6.5 (0.5; 12.5)	0.182	15.3 (6.2; 24.4)	5.9 (0.4; 11.4)	0.064	−10.6 (−26.7; 5.5)	−9.4 (−19.4; 0.6)	0.535
Abdominal pain frequency	7.1 (0.8; 13.3)	7.1 (0.8; 13.3)	0.999	12.9 (6; 19.9)	5.3 (0.8; 9.8)	0.067	0 (−0.9; 9.4)	−7.6 (−15.9; 0.6)	0.280
Bloating	27.1 (14.2; 39.9)	18.8 (6.9; 30.7)	0.105	25.9 (13.8; 37.9)	26.5 (15; 38)	0.894	−8.2 (−18.4; 1.9)	0.6 (−8.6; 9.8)	0.162
Bowel satisfaction	79.4 (66.4; 92.5)	78.8 (64.2; 93.5)	0.922	68.2 (53; 83.5)	63.5 (48.5; 78.5)	0.512	−0.6 (−13.1; 11.9)	−4.7 (−19.6; 10.2)	0.518
Abdominal heaviness	30 (16.5; 43.5)	18.2 (5.7; 30.7)	0.053	30 (17; 43)	30.6 (18.9; 42.3)	0.913	−11.8 (−23.7; 0.2)	0.6 (−10.7; 11.9)	0.138
Quality of life	24.4 (12; 36.9)	13.5 (3.1; 24)	0.064	22.4 (8.4; 36.3)	19.4 (8; 30.8)	0.673	−10.9 (−22.4; 0.7)	−2.9 (−17.5; 11.6)	0.176

General linear model for repeated measurements. Data are reported as geometric mean and 95% confidence interval.

† *p* value calculated using the Wilcoxon signed-ranks test.

° *p* value calculated using the Mann–Whitney test.

GAI, Global Assessment of Improvement Scale; SSS, Symptom Severity Scale. Statistically significant *p*-values are shown in bold.

### Characterization of gut microbiota composition and function

**Figure 1:**
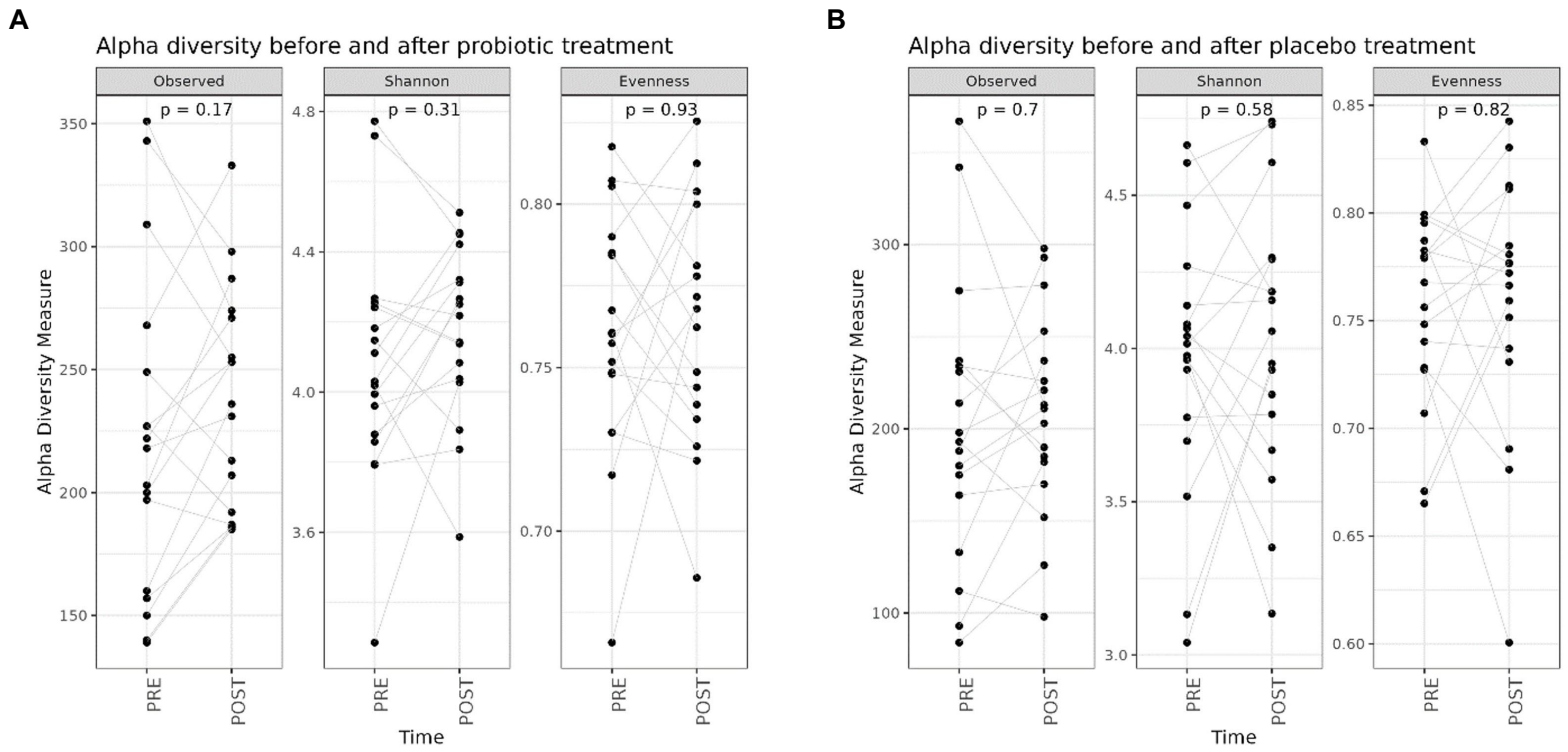
Box plots reporting alpha diversity indices (Observed ASV richness, Shannon index, Pielou’s evenness) pre- (*n* = 20) and post-probiotic (*n* = 20) **(A)** and pre- (*n* = 20) and post-placebo (n = 20) **(B)** treatments. Lines link paired samples and statistical differences were assessed using the Wilcoxon signed-rank test. *p*-values less than 0.05 were considered statistically significant.

First, we evaluated if the probiotic and the placebo treatments determined an impact on the GM composition (Supplementary Figure 2) and, as reported in Figure 1, no significant differences were reported on the different alpha diversity indices after both interventions (probiotic vs. placebo).

**Figure 2:**
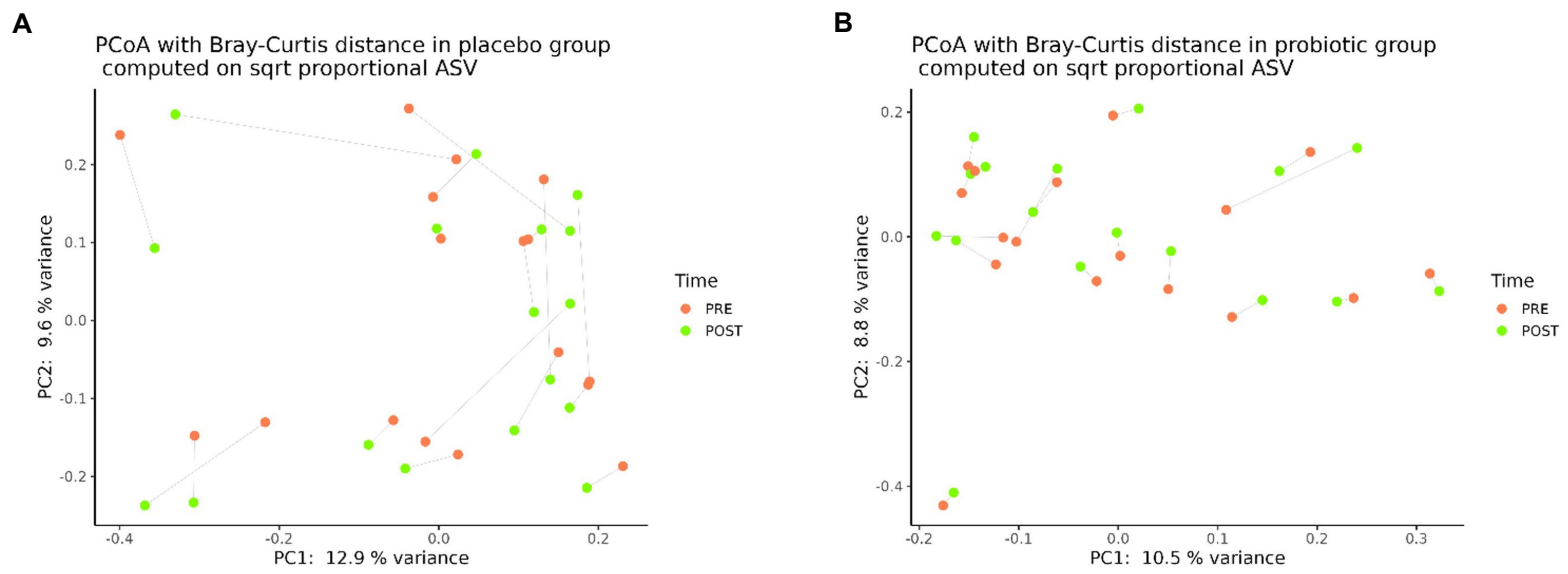
Principal coordinates analysis (PCoA), according to the Bray-Curtis beta-diversity metric, of pre- (*n* = 20) and post- (*n* = 20) placebo samples **(A)** and pre- (*n* = 20) and post- (*n* = 20) probiotic samples **(B)**.

Likewise, regarding the GM composition, beta-diversity analysis assessed through the Bray–Curtis dissimilarity metric, did not highlight any clear separation between pre- and post-probiotic samples (PERMANOVA, *p* = 0.9999) (Figure 2A) or among the pre- and post-placebo samples (PERMANOVA, *p* = 0.9995) (Figure 2B).

Furthermore, to evaluate the impact of both interventions on microbial abundances, we performed the differential analysis at all taxonomic ranks.

**Figure 3:**
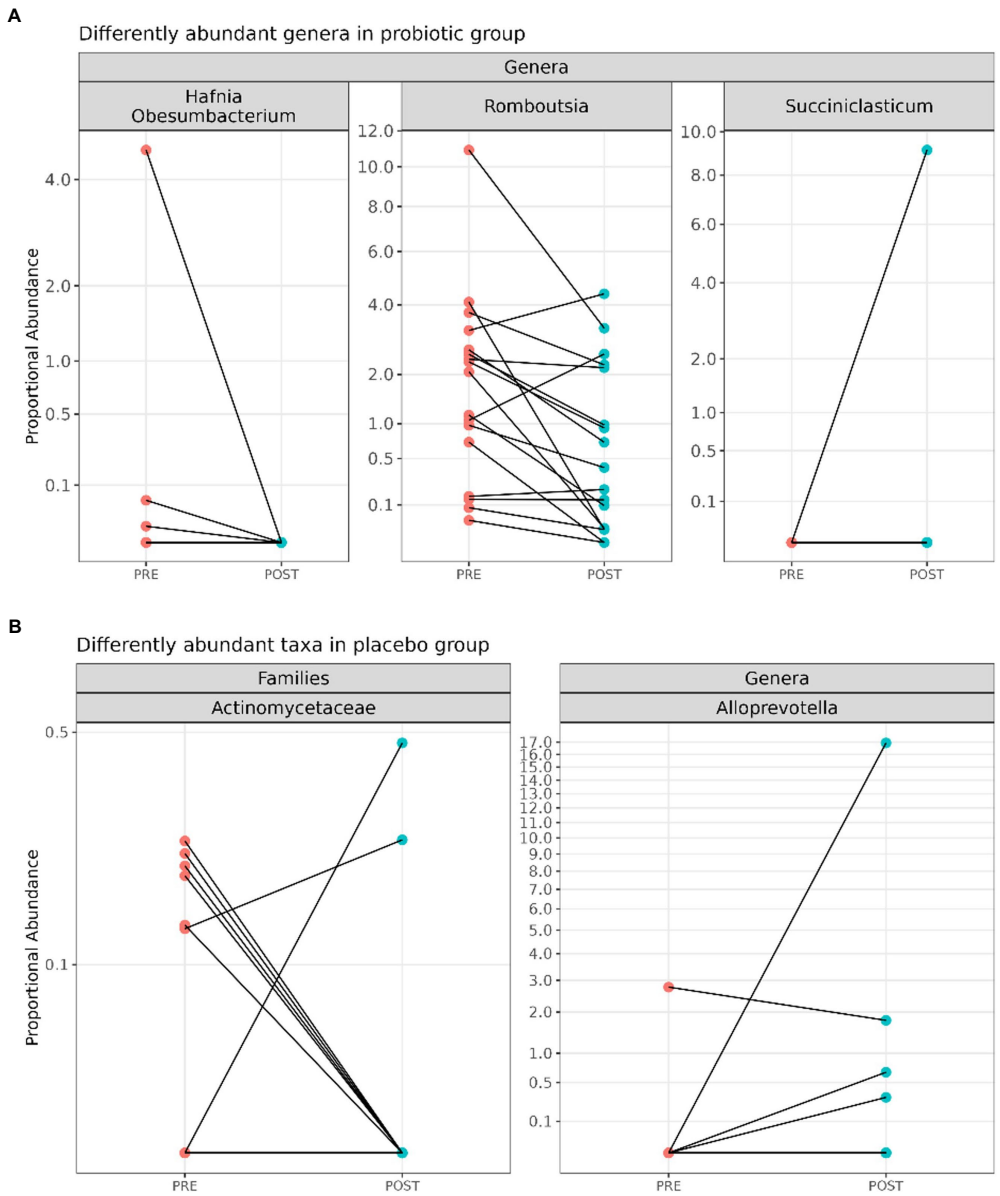
Line plots displaying significant differentially abundant taxa between pre- (n = 20) and post-(*n* = 20) probiotic samples **(A)** and among pre- (*n* = 20) and post- (*n* = 20) placebo **(B)** samples.

In detail, probiotic supplementation resulted in the decrease of the genera *Hafnia-Obesumbacterium* (log2FC = 27.3; adj. *p* = 6.19e-18) and *Romboutsia* (log2FC = 1.95; adj.*p* = 0.02) and in the increase of the genus *Succiniclasticum* (log2FC = 20.1, adj.*p* = 1.09e-9) (Figure 3A).

On the other hand, as shown in Figure 3B, the reduction of the family Actinomycetaceae (log2FC = 11.1; adj. *p* = 0.01) and the increase of the genus *Alloprevotella* (log2FC = 13.5; adj. p = 0.01) were reported after the placebo administration.

The obesity condition was associated with an increased *Firmicutes/Bacteroidota* (F/B) ratio. In this context, our results put in evidence a close to significance decrease (*p* = 0.09) in the F/B ratio in the probiotic group’s subjects at the end of the intervention with respect to the baseline and respect to the placebo group (*p* = 0.78) (Supplementary Figure 3). Accordingly, the probiotic administration maintains a lower F/B ratio, extending previously obtained results and strengthening the probiotics’ use in obesity management.

In addition, we assessed the fecal SCFAs and MCFAs abundances before and after both treatments, and the results are reported in Table 5. In detail, no statistically significant differences were reported after the probiotic administration, while placebo treatment resulted in significant (*p* < 0.05) increased levels of hexanoic (+0.22%) and heptanoic acids (+0.08%).

**Table 5:** SCFAs and MCFAs variation according to the dietary interventions.

Variable	Probiotic pre	Probiotic post	*p* value^†^	Placebo pre	Placebo post	*p* value^†^	Change Probiotic	Change Placebo	*p* value^°^
*SCFAs (%)*									
Acetic acid	52.44 (7.03)	52.36 (4.36)	0.919	53.58 (9.23)	51.67 (4.13)	0.448	−0.09 (8.37)	−1.91 (10.53)	0.536
Propionic acid	18.68 (3.34)	19.90 (4.60)	0.177	18.40 (3.82)	17.57 (3.71)	0.570	1.23 (4.55)	−0.83 (5.71)	0.183
Butyric acid	16.37 (7.54)	15.72 (4.52)	0.888	15.22 (5.93)	15.69 (4.20)	0.962	−0.65 (5.01)	0.47 (5.18)	0.727
Isobutyric acid	2.00 (1.01)	2.14 (1.25)	0.506	2.33 (1.78)	2.48 (1.62)	0.493	0.13 (1.01)	0.15 (0.73)	0.886
2-Methylbutyric acid	1.39 (1.04)	1.50 (0.88)	0.386	1.70 (1.92)	1.93 (1.28)	0.299	0.11 (0.83)	0.23 (0.90)	0.787
Isovaleric acid	1.45 (0.88)	1.58 (1.12)	0.386	1.75 (1.87)	1.92 (1.18)	0.434	0.13 (0.80)	0.17 (0.80)	0.936
Valeric acid	2.98 (1.24)	3.11 (0.92)	0.212	2.94 (0.81)	2.85 (1.45)	0.962	0.13 (0.71)	−0.09 (1.35)	0.787
*MCFAs (%)*									
Hexanoic acid	0.72 (1.15)	0.80 (0.90)	0.586	0.57 (0.55)	0.79 (0.91)	**0.035**	0.08 (1.04)	0.22 (0.39)	0.505
Isohexanoic acid	0.04 (0.04)	0.03 (0.14)	0.481	0.03 (0.03)	0.03 (0.03)	0.722	−0.01 (0.04)	0.00 (0.01)	0.871
Heptanoic acid	0.12 (0.19)	0.14 (0.16)	0.355	0.09 (0.14)	0.17 (0.17)	**0.033**	0.02 (0.15)	0.08 (0.15)	0.535
Octanoic acid	0.03 (0.02)	0.02 (0.02)	0.563	0.03 (0.03)	0.03 (0.03)	0.609	0.00 (0.01)	0.00 (0.01)	0.895
Nonanoic acid	0.00 (0.00)	0.00 (0.00)	0.071	0.00 (0.00)	0.00 (0.00)	0.109	0.00 (0.00)	0.00 (0.00)	0.508
Decanoic acid	0.01 (0.00)	0.01 (0.00)	0.255	0.01 (0.01)	0.01 (0.00)	0.773	0.00 (0.01)	0.00 (0.01)	0.460
Dodecanoic acid	0.01 (0.01)	0.01 (0.01)	0.560	0.02 (0.02)	0.03 (0.04)	0.219	0.00 (0.01)	0.00 (0.02)	0.488

Data are presented as median percentage (interquartile range, IQR).

† *p* value calculated using the Wilcoxon signed-ranks test.

° *p* value calculated using the Mann–Whitney test. SCFAs, short-chain fatty acids; MCFAs, medium-chain fatty acids. Statistically significant *p*-values are shown in bold.

## Discussion

Obesity is well known as a complex and multifactorial disease and its incidence is steadily increasing, with pandemic levels being already reached because of easy access to high-energy, processed foods and a sedentary lifestyle (19). In recent years, obesity pathogenesis has been closely linked to variations in GM function and structure; hence, considering that the modulation of gut bacteria composition resulted in changes in body mass index, probiotics have been proposed as a novel promising therapeutic strategy to treat/prevent obesity (20).

Therefore, in this study, we have evaluated the effects of a novel probiotic formulation based on *Lactiplantibacillus plantarum* IMC 510® on anthropometric and biochemical parameters, GM composition and function, and gastrointestinal and general symptoms of overweight/obese subjects.

First, regarding the anthropometric parameters, we found that probiotic supplementation resulted in a slight reduction in body weight, BMI, WHt ratio, and fat mass, confirming our previous findings that documented a clear significant reduction in body weight gain, BMI, and white adipose tissue weight in obese rats after 84 days of *Lactiplantibacillus plantarum* IMC 510® supplementation (15) and in human subjects after 3 months of *Lactiplantibacillus plantarum* IMC 510® administration (16).

More specifically, it has been documented that *Lactiplantibacillus plantarum* strains alleviated obesity in mice by activating the PPARα/CPT1α pathway, downregulating SREBP-1 and tDGAT1 mRNA expression levels or by increasing the expression of bile secretion-related genes cholesterol 7α-hydroxylase (Cyp7α1) (21). Moreover*, Lactiplantibacillus plantarum* strains have led to a reduction of body weight and fat volume in high-fat diet-fed obese mice downregulating the lipogenic genes PPARγ, HSL, SCD-1, and FAT/CD36 (22).

In general, although a recent meta-analysis reported that the supplementation of different probiotics species through capsules or added to foods has determined a significantly larger reduction in body weight and fat percentage (23), other findings suggested that the probiotic effect on body weight is strain specific and more research is needed to clarify mechanisms by which certain microorganisms help weight loss (13).

In fact, in addition to the well noted anti-obesogenic effects of different *Lactobacillus* and *Bifidobacterium* strains (20), several “next-gen” probiotics are currently under study for their capability to reduce body weight (24).

For instance, oral treatment of high-fat diet-fed mice with *Parabacteroides goldsteinii* was associated with reduced obesity enhanced intestinal integrity and reduced levels of inflammation and insulin resistance (25), while the daily administration of *Saccharomyces cerevisiae* var. boulardii to leptin-resistant obese and type 2 diabetic mice led to a reduction of body weight, fat mass, hepatic steatosis, and inflammatory tone (26).

Moreover, also *Akkermansia muciniphila* has been recently characterized as a beneficial player in body metabolism because of its capability to reduce obesity by regulating metabolism and energy hemostasis and improving insulin sensitivity and glucose hemostasis (27).

In addition, in accordance with Coman et al. (16), probiotic supplementation has also determined a significant decrease in the waist circumference of volunteers compared to placebo. Waist circumference and WHtR are used in clinical practice as markers of visceral adiposity, tightly associated with obesity and related metabolic diseases, and the reduction of these two parameters provides important cardiovascular benefits to obese individuals (28). Waist circumference and WHt ratio, and not BMI, are crucial measures of obesity severity and are more closely associated with obesity-related metabolic diseases. The probiotics appeared to be able to alter fat distribution without significantly changing the body weight in obese/overweight subjects, which is reflected in the reduced waist circumference and the WHt ratio meaning the probiotics’ use seems to be beneficial to this kind of patient.

The 3 months supplementation seems to be the starting point of the trend in observing positive effects on body weight and/or fat mass (waist circumference and WHt ratio) by using probiotics. It should be noted that a decrease of ≥1% in initial body weight in a period of 3 months is clinically relevant (29) and this would be associated with a significant reduction in some cardiovascular risk factors, such as a decrease in blood glucose, as revealed by our data. Therefore, compared to placebo, probiotic intervention determined a significant reduction in blood glucose, while no significant changes were for other blood parameters. Consistent with our findings, a meta-analysis performed by Kocsis and colleagues reported that probiotic supplementation, although consisting of both the administration of one bacterial species or a combination of more than one strain, determined a significant reduction in fasting plasma glucose levels in patients with type 2 diabetes mellitus (30).

Furthermore, changes in gastrointestinal and systemic symptoms have been evaluated through validated questionnaires and the *Lactiplantibacillus plantarum* IMC 510® supplementation resulted in a slight, but not significant improvement of abdominal heaviness and the interference of gastrointestinal symptoms with quality of life, while, on the other hand, a slight reduction in intensity and frequency of abdominal pain was observed after placebo intervention. According to our findings, many studies have reported the beneficial effects of probiotics on bloating scores and abdominal pain (31–33), but, in general, probiotics enhance the host’s well-being: (i) protecting against pathogenic bacteria, (ii) stabilizing the gut permeability, (iii) increasing the intestinal barrier functionality, (iv) normalizing bowel movements, and (v) reducing visceral hypersensitivity (34).

In addition, considering that probiotic supplementation is mainly used to restore the GM, we have evaluated if *Lactiplantibacillus plantarum* IMC 510® administration determined microbial compositional modifications in overweight/obese individuals. However, both probiotic and placebo interventions did not significantly modify either the structure or the different microbial alpha diversity indices and only a slight increase in observed ASV richness was reported after *Lactiplantibacillus plantarum* IMC 510® treatment. In agreement with our findings, although various research has highlighted a lower alpha diversity in overweight/obese compared to normal-weight humans, other studies reported that probiotics based on *Lactobacillus* and *Bifidobacterium* strains did not significantly impact alpha diversity in overweight/obese individuals (35, 36).

On the other hand, some significant variations have been reported by differential analysis performed at all taxonomic ranks. *Lactiplantibacillus plantarum* IMC 510® supplementation determined the significant increase of *Succiniclasticum* spp. and the reduction of *Hafnia-Obesumbacterium* and *Romboutsia* genera. Interestingly, a lower abundance of *Romboutsia* spp. has been recently associated with lower LDL-cholesterol levels and a higher intake of fibers (37).

Moreover, we have assessed the fecal SCFAs and MCFAs abundances and, in line with the absence of remarkable compositional modifications in GM, no statistically significant differences were reported after the probiotic administration. In general, SCFAs contribute to the regulation of host homeostasis by acting as potent activators of G-protein-coupled receptors that are expressed in intestinal, liver, pancreatic tissues, and white adipose tissues, promoting hepatic lipogenesis and an energy imbalance (38). In addition, experimental studies in both obese subjects and animal models have suggested that SCFAs could increase fat oxidation and energy expenditure and decrease lipolysis (39). Anyway, further investigations about the metabolic role of SCFAs are needed to resolve the paradox between the high fecal SCFAs’ abundances in obese subjects and their lack of efficient satiety signal, possibly determined by the attenuated binding of SCFAs to their receptors which could occur during obesity (40).

On the contrary, placebo treatment resulted in significant increase in levels of hexanoic and heptanoic acids. MCFAs are known as pro-inflammatory mediators, especially due to their ability to enhance Th1 and Th17 cell differentiation; moreover, these T cells are involved in the persistence of the gut inflammation that commonly characterizes obese subjects (41, 42).

Of course, the present study has some limitations, such as the restricted sample size, the short duration of probiotic supplementation, and the low taxonomical resolution of 16S rRNA sequencing. However, our data clearly reported that the novel probiotic strain *Lactiplantibacillus plantarum* IMC 510® showed, in addition to the increase of some beneficial gut bacteria, lowering effects on body weight, waist circumference, fasting glucose levels, and gastrointestinal symptoms of obese individuals. Surely, future studies investigating if the administration of a low-calorie dietary intervention in addition to the association of a dietary intervention to the *Lactiplantibacillus plantarum* IMC 510® supplementation could be useful to increase the probiotic beneficial effects on overweight/obese subjects are needed. To conclude, although the best strategies to prevent obesity remain the control of diet and lifestyle factors and further studies about the administration of the probiotics for GM modulation are needed, *Lactiplantibacillus plantarum* IMC 510® supplementation could represent a future and encouraging strategy for the prevention or treatment of obesity.

## Data availability statement

The data presented in this study are deposited in the NCBI Gene Expression Omnibus (GEO) repository, accession number GSE222755.

## Ethics statement

The studies involving human participants were reviewed and approved by Ethics Committee of the Tuscany Region, Careggi University Hospital. The patients/participants provided their written informed consent to participate in this study.

## Author contributions

MMC and MCV conceived and designed the study. GP, MMC, and SB drafted the paper. SB, GN, ER, and LC acquired experimental data of microbiota. GP, MD, BC, and SL were involved in subjects’ enrolment. SB, MP, and GB performed gas chromatography-mass spectrometer analysis. LDG and MR performed bioinformatic and statistical analysis of microbiota. GP, MMC, SB, MD, ER, BC, SL, MP, LDG, GB, MR, and MCV analyzed and interpreted the data. MMC, MCV, FS, and AA critically revised the paper. All authors contributed to the article and approved the submitted version.

## Funding

This research was supported by MICAfrica Project, which is funded by The European Commission Programme 2020-WIDE SPREAD-05-2020-Twinning Grant Agreement, No. 952583.

## Conflict of interest

MMC and MCV were employed by Synbiotec S.r.l.

The remaining authors declare that the research was conducted in the absence of any commercial or financial relationships that could be construed as a potential conflict of interest.

## Publisher’s note

All claims expressed in this article are solely those of the authors and do not necessarily represent those of their affiliated organizations, or those of the publisher, the editors and the reviewers. Any product that may be evaluated in this article, or claim that may be made by its manufacturer, is not guaranteed or endorsed by the publisher.
