# Supplementary material for: Effects of the probiotic Lactiplantibacillus plantarum IMC 510® on body composition, biochemical parameters, gut microbiota composition and function, and clinical symptoms of overweight/obese subjects
Source: Front Nutr. 2023 Apr 11;10:1142527. doi: 10.3389/fnut.2023.1142527 (PMC10130646; doi:10.3389/fnut.2023.1142527)
Supplement: Supplementary file 1 [file Data_Sheet_1.docx]

Supplementary Material

Effects of the probiotic Lactiplantibacillus plantarum IMC 510® on body composition, biochemical parameters, gut microbiota composition and function and clinical symptoms of overweight/obese subjects

Giuditta Pagliai^†^, Maria Magdalena Coman^†^, Simone Baldi^†^, Monica Dinu, Giulia Nannini, Edda Russo, Lavinia Curini, Barbara Colombini, Sofia Lotti, Marco Pallecchi, Leandro Di Gloria, Gianluca Bartolucci, Matteo Ramazzotti, Maria Cristina Verdenelli, Francesco Sofi^*^, Amedeo Amedei^*^

*** Correspondence:** Francesco Sofi: [francesco.sofi@unifi.it](mailto:francesco.sofi@unifi.it); Amedeo Amedei: [amedeo.amedei@unifi.it](mailto:amedeo.amedei@unifi.it)

# Supplementary Figures

Assessed for eligibility (n=56)

Excluded (n=16)

♦  Not meeting inclusion criteria (n=12)

♦  Declined to participate (n=3)

♦  Other reasons (n=1)

Analysed (n=20)
♦ Excluded from analysis (n=0)

Lost to follow-up (n=0)

Discontinued intervention (n=0)

Allocated to probiotic group (n=20)

♦ Received allocated intervention (n=20)

♦ Did not receive allocated intervention (n=0)

Lost to follow-up (n=0)

Discontinued intervention (n=0)

Allocated to placebo group (n=20)

♦ Received allocated intervention (n=20)

♦ Did not receive allocated intervention (n=0)

Analysed (n=20)
♦ Excluded from analysis (n=0)

## Allocation

## Analysis

## Follow-Up

Randomized (n=40)

## Enrollment

**Supplementary Figure 1.** CONSORT flow diagram.


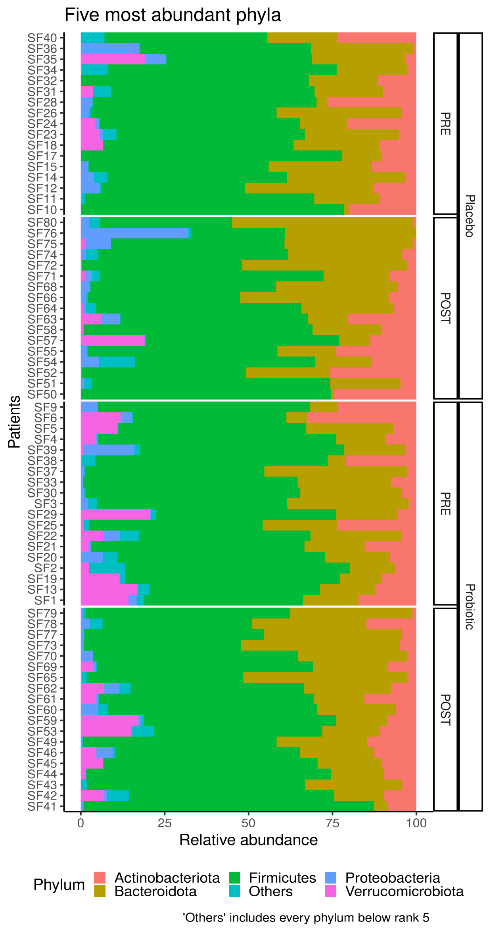

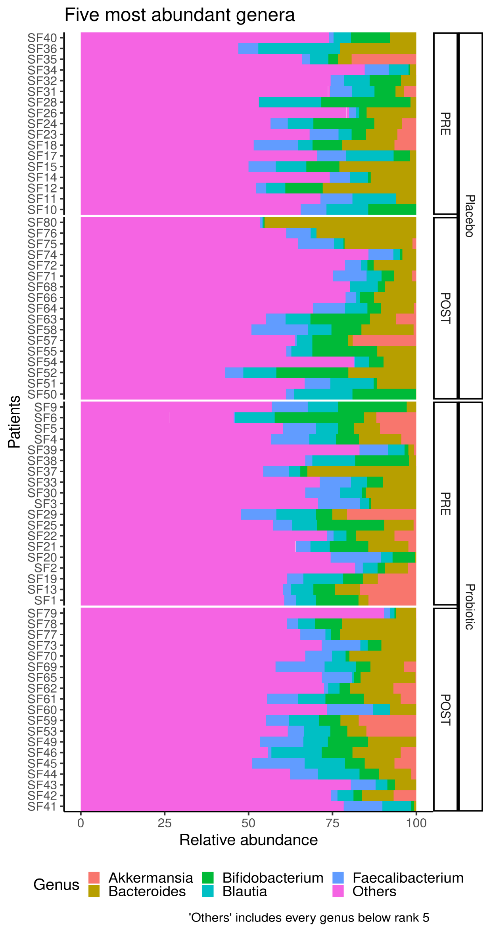


A)

B)

**Supplementary Figure 2**. Stacked boxplots of microbial composition between pre- and post- placebo samples and pre- and post- probiotic samples at both phylum (A) and genera (B) levels.


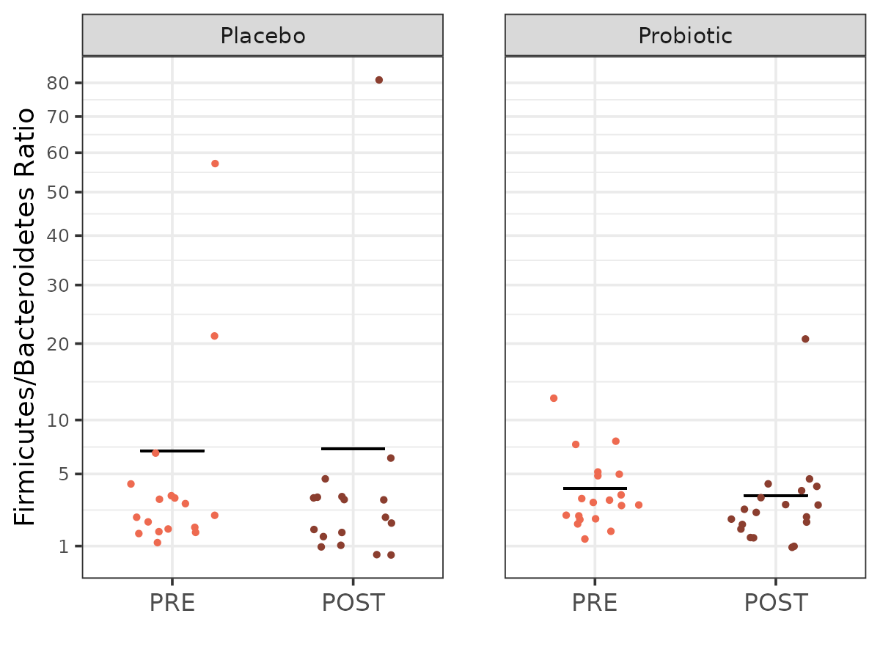


A)

B)

**Supplementary Figure 3.** Firmicutes/Bacteroidetes (F/B) ratio assessed pre- and post- placebo samples (A) and pre- and post- probiotic samples (B).

# Supplementary Table 1. Number of obtained and processed reads for each sample.

| sample-id | input | filtered | % input passed filter | denoised | merged | % input merged | non-chimeric | % input non-chimeric |
| --- | --- | --- | --- | --- | --- | --- | --- | --- |
| ID2371-1-SF1-A1-A01 | 97784 | 74716 | 76.41 | 71614 | 63618 | 65.06 | 42634 | 43.6 |
| ID2371-10-SF10-A1-B02 | 61495 | 47418 | 77.11 | 46016 | 41918 | 68.16 | 20640 | 33.56 |
| ID2371-11-SF11-A1-C02 | 70843 | 55344 | 78.12 | 53823 | 48620 | 68.63 | 30872 | 43.58 |
| ID2371-12-SF12-A1-D02 | 82492 | 63050 | 76.43 | 60232 | 53520 | 64.88 | 30523 | 37 |
| ID2371-13-SF13-A1-E02 | 89396 | 69115 | 77.31 | 66272 | 58497 | 65.44 | 34074 | 38.12 |
| ID2371-14-SF14-A1-F02 | 68172 | 51848 | 76.05 | 48303 | 40357 | 59.2 | 25031 | 36.72 |
| ID2371-15-SF15-A1-G02 | 63016 | 49264 | 78.18 | 47218 | 41879 | 66.46 | 23137 | 36.72 |
| ID2371-17-SF17-A1-A03 | 75656 | 58288 | 77.04 | 55410 | 45864 | 60.62 | 25003 | 33.05 |
| ID2371-18-SF18-A1-B03 | 67026 | 51766 | 77.23 | 49237 | 41989 | 62.65 | 21880 | 32.64 |
| ID2371-19-SF19-A1-C03 | 78649 | 60981 | 77.54 | 57368 | 48845 | 62.11 | 28322 | 36.01 |
| ID2371-2-SF2-A1-B01 | 86551 | 66605 | 76.95 | 61751 | 51801 | 59.85 | 30388 | 35.11 |
| ID2371-20-SF20-A1-D03 | 69319 | 51317 | 74.03 | 48031 | 40636 | 58.62 | 26649 | 38.44 |
| ID2371-21-SF21-A1-E03 | 77555 | 59659 | 76.92 | 56659 | 48465 | 62.49 | 27766 | 35.8 |
| ID2371-22-SF22-A1-F03 | 76049 | 57807 | 76.01 | 51786 | 41158 | 54.12 | 28348 | 37.28 |
| ID2371-23-SF23-A1-G03 | 75701 | 59111 | 78.08 | 53607 | 43120 | 56.96 | 29725 | 39.27 |
| ID2371-24-SF24-A1-H03 | 72126 | 55573 | 77.05 | 52740 | 45723 | 63.39 | 28249 | 39.17 |
| ID2371-25-SF25-A1-A04 | 77016 | 59325 | 77.03 | 55905 | 46837 | 60.81 | 27164 | 35.27 |
| ID2371-26-SF26-A1-B04 | 84892 | 64376 | 75.83 | 59658 | 50739 | 59.77 | 31202 | 36.75 |
| ID2371-28-SF28-A1-D04 | 87813 | 64265 | 73.18 | 63150 | 59131 | 67.34 | 27724 | 31.57 |
| ID2371-29-SF29-A1-E04 | 111460 | 67778 | 60.81 | 65112 | 56911 | 51.06 | 38495 | 34.54 |
| ID2371-3-SF3-A1-C01 | 82261 | 62002 | 75.37 | 56424 | 44417 | 54 | 27576 | 33.52 |
| ID2371-30-SF30-A1-F04 | 63513 | 48656 | 76.61 | 45458 | 36542 | 57.53 | 20988 | 33.05 |
| ID2371-31-SF31-A1-G04 | 84839 | 65967 | 77.76 | 61742 | 51466 | 60.66 | 32754 | 38.61 |
| ID2371-32-SF32-A1-H04 | 77047 | 59112 | 76.72 | 56101 | 48244 | 62.62 | 28205 | 36.61 |
| ID2371-33-SF33-A1-A05 | 106873 | 83491 | 78.12 | 80262 | 70283 | 65.76 | 37362 | 34.96 |
| ID2371-34-SF34-A1-B05 | 165824 | 128513 | 77.5 | 122257 | 104304 | 62.9 | 59716 | 36.01 |
| ID2371-35-SF35-A1-C05 | 122912 | 96781 | 78.74 | 93948 | 87134 | 70.89 | 59230 | 48.19 |
| ID2371-36-SF36-A1-D05 | 167976 | 130267 | 77.55 | 127006 | 116881 | 69.58 | 65832 | 39.19 |
| ID2371-37-SF37-A1-E05 | 186048 | 146304 | 78.64 | 140826 | 122463 | 65.82 | 68750 | 36.95 |
| ID2371-38-SF38-A1-F05 | 113209 | 87204 | 77.03 | 83972 | 73313 | 64.76 | 37030 | 32.71 |
| ID2371-39-SF39-A1-G05 | 98275 | 75277 | 76.6 | 70486 | 58800 | 59.83 | 38188 | 38.86 |
| ID2371-4-SF4-A1-D01 | 79629 | 60501 | 75.98 | 58102 | 50565 | 63.5 | 25554 | 32.09 |
| ID2371-40-SF40-A1-H05 | 77140 | 58071 | 75.28 | 54895 | 47849 | 62.03 | 31588 | 40.95 |
| ID2371-41-SF41-A1-A06 | 134259 | 104625 | 77.93 | 101637 | 91208 | 67.93 | 45036 | 33.54 |
| ID2371-42-SF42-A1-B06 | 169176 | 130458 | 77.11 | 124713 | 109796 | 64.9 | 66401 | 39.25 |
| ID2371-43-SF43-A1-C06 | 121694 | 91114 | 74.87 | 85722 | 72720 | 59.76 | 43838 | 36.02 |
| ID2371-44-SF44-A1-D06 | 179268 | 137421 | 76.66 | 134134 | 122387 | 68.27 | 66371 | 37.02 |
| ID2371-45-SF45-A1-E06 | 169785 | 131043 | 77.18 | 126585 | 113847 | 67.05 | 69305 | 40.82 |
| ID2371-46-SF46-A1-F06 | 86721 | 64276 | 74.12 | 61365 | 54458 | 62.8 | 33727 | 38.89 |
| ID2371-49-SF49-A1-A07 | 103471 | 79461 | 76.8 | 76227 | 65613 | 63.41 | 36112 | 34.9 |
| ID2371-5-SF5-A1-E01 | 66845 | 51218 | 76.62 | 48240 | 40010 | 59.85 | 24971 | 37.36 |
| ID2371-50-SF50-A1-B07 | 112145 | 85765 | 76.48 | 84251 | 79375 | 70.78 | 43146 | 38.47 |
| ID2371-51-SF51-A1-C07 | 105593 | 82059 | 77.71 | 78766 | 69060 | 65.4 | 41005 | 38.83 |
| ID2371-52-SF52-A1-D07 | 180534 | 136657 | 75.7 | 133068 | 120533 | 66.76 | 58168 | 32.22 |
| ID2371-53-SF53-A1-E07 | 115954 | 88555 | 76.37 | 84360 | 73342 | 63.25 | 45548 | 39.28 |
| ID2371-54-SF54-A1-F07 | 68200 | 50937 | 74.69 | 47592 | 41063 | 60.21 | 27509 | 40.34 |
| ID2371-55-SF55-A1-G07 | 79789 | 61157 | 76.65 | 58355 | 51111 | 64.06 | 28143 | 35.27 |
| ID2371-57-SF57-A1-A08 | 104369 | 79992 | 76.64 | 76953 | 69736 | 66.82 | 38803 | 37.18 |
| ID2371-58-SF58-A1-B08 | 115877 | 89745 | 77.45 | 86652 | 75867 | 65.47 | 37420 | 32.29 |
| ID2371-59-SF59-A1-C08 | 85836 | 66848 | 77.88 | 63213 | 54273 | 63.23 | 33012 | 38.46 |
| ID2371-6-SF6-A1-F01 | 59930 | 42891 | 71.57 | 41020 | 36412 | 60.76 | 20123 | 33.58 |
| ID2371-60-SF60-A1-D08 | 159709 | 118185 | 74 | 113047 | 98013 | 61.37 | 58221 | 36.45 |
| ID2371-61-SF61-A1-E08 | 207593 | 160965 | 77.54 | 155956 | 139810 | 67.35 | 73906 | 35.6 |
| ID2371-62-SF62-A1-F08 | 50630 | 37702 | 74.47 | 34055 | 27864 | 55.03 | 20562 | 40.61 |
| ID2371-63-SF63-A1-G08 | 86376 | 67029 | 77.6 | 63853 | 55051 | 63.73 | 31206 | 36.13 |
| ID2371-64-SF64-A1-H08 | 97044 | 74765 | 77.04 | 69871 | 55879 | 57.58 | 35901 | 36.99 |
| ID2371-65-SF65-A1-A09 | 71315 | 55341 | 77.6 | 53406 | 47894 | 67.16 | 34526 | 48.41 |
| ID2371-66-SF66-A1-B09 | 199617 | 151524 | 75.91 | 145477 | 130357 | 65.3 | 88948 | 44.56 |
| ID2371-68-SF68-A1-D09 | 102510 | 78056 | 76.14 | 75388 | 68814 | 67.13 | 46358 | 45.22 |
| ID2371-69-SF69-A1-E09 | 90569 | 70514 | 77.86 | 67803 | 60414 | 66.7 | 36836 | 40.67 |
| ID2371-70-SF70-A1-F09 | 71822 | 55332 | 77.04 | 53453 | 48226 | 67.15 | 35142 | 48.93 |
| ID2371-71-SF71-A1-G09 | 55551 | 43306 | 77.96 | 40613 | 35840 | 64.52 | 31262 | 56.28 |
| ID2371-72-SF72-A1-H09 | 74695 | 57998 | 77.65 | 54949 | 48953 | 65.54 | 35853 | 48 |
| ID2371-73-SF73-A1-A09 | 94597 | 72987 | 77.16 | 70652 | 64633 | 68.32 | 41932 | 44.33 |
| ID2371-74-SF74-A1-B10 | 58148 | 43299 | 74.46 | 39510 | 32202 | 55.38 | 24673 | 42.43 |
| ID2371-75-SF75-A1-C10 | 67179 | 53258 | 79.28 | 50545 | 44117 | 65.67 | 25565 | 38.06 |
| ID2371-76-SF76-A1-D10 | 117369 | 89961 | 76.65 | 87932 | 83319 | 70.99 | 51890 | 44.21 |
| ID2371-77-SF77-A1-E10 | 87908 | 68464 | 77.88 | 65128 | 56930 | 64.76 | 38496 | 43.79 |
| ID2371-78-SF78-A1-F10 | 78192 | 58925 | 75.36 | 55484 | 48502 | 62.03 | 31256 | 39.97 |
| ID2371-79-SF79-A1-G10 | 94467 | 72237 | 76.47 | 68907 | 61397 | 64.99 | 45861 | 48.55 |
| ID2371-80-SF80-A1-H10 | 77019 | 57345 | 74.46 | 55706 | 51952 | 67.45 | 34475 | 44.76 |
| ID2371-9-SF9-A1-A02 | 86114 | 66007 | 76.65 | 63455 | 54918 | 63.77 | 28860 | 33.51 |
